# Discovery of serum biomarkers for pancreatic adenocarcinoma using proteomic analysis

**DOI:** 10.1038/sj.bjc.6605764

**Published:** 2010-06-29

**Authors:** A Xue, C J Scarlett, L Chung, G Butturini, A Scarpa, R Gandy, S R Wilson, R C Baxter, R C Smith

**Affiliations:** 1 Department of Surgery, University of Sydney, Royal North Shore Hospital, St Leonards, New South Wales 2065, Australia; 2 Kolling Institute of Medical Research, University of Sydney, Royal North Shore Hospital, St Leonards, New South Wales 2065, Australia; 3 Surgical and Anaesthesiological Department, University of Verona, Piazzale La Scuro, Verona 1037134, Italy; 4 Pathology Department, University of Verona, Piazzale La Scuro, Verona 1037134, Italy; 5 Mathematical Sciences Institute, Australian National University, Canberra, Australian Capital Territory 0200, Australia

**Keywords:** pancreatic adenocarcinoma, proteomics, serum biomarkers, apolipoproteins

## Abstract

**Background and aims:**

The serum/plasma proteome was explored for biomarkers to improve the diagnostic ability of CA19-9 in pancreatic adenocarcinoma (PC).

**Methods:**

A Training Set of serum samples from 20 resectable and 18 stage IV PC patients, 54 disease controls (DCs) and 68 healthy volunteers (HVs) were analysed by surface-enhanced laser desorption and ionisation time-of-flight mass spectrometry (SELDI-TOF MS). The resulting protein panel was validated on 40 resectable PC, 21 DC and 19 HV plasma samples (Validation-1 Set) and further by ELISA on 33 resectable PC, 28 DC and 18 HV serum samples (Validation-2 Set). Diagnostic panels were derived using binary logistic regression incorporating internal cross-validation followed by receiver operating characteristic (ROC) analysis.

**Results:**

A seven-protein panel from the training set PC *vs* DC and from PC *vs* HV samples gave the ROC area under the curve (AUC) of 0.90 and 0.90 compared with 0.87 and 0.91 for CA19-9. The AUC was greater (0.97 and 0.99, *P*<0.05) when CA19-9 was added to the panels and confirmed on the validation-1 samples. A simplified panel of apolipoprotein C-I (ApoC-I), apolipoprotein A-II (ApoA-II) and CA19-9 was tested on the validation-2 set by ELISA, in which the ROC AUC was greater than that of CA19-9 alone for PC *vs* DC (0.90 *vs* 0.84) and for PC *vs* HV (0.96 *vs* 0.90).

**Conclusions:**

A simplified diagnostic panel of CA19-9, ApoC-I and ApoA-II improves the diagnostic ability of CA19-9 alone and may have clinical utility.

Diagnostic serum biomarkers for pancreatic cancer are unsatisfactory (Honda *et al*, 2005), although many have been investigated (Grote and Logsdon, 2007). CA19-9, the ‘gold standard’ (Goonetilleke and Siriwardena, 2007), has a 79% (range reported in the literature 70–90%) sensitivity and 82% (68–91%) specificity. Although grossly elevated CA19-9 predicts unresectable disease (Maithel *et al*, 2008) and prognosis for chemoradiotherapy (Berger *et al*, 2008), 10–15% of patients cannot produce CA19-9 because of Lewis-negative genotype (Kawai *et al*, 2008). In addition, serum CA19-9 is elevated in other malignancies and benign disorders (Kim *et al*, 2004). Recently, the necessity for a multivariate serum marker has been proposed (Grote and Logsdon, 2007).

The low-molecular-weight proteome (<10 kDa) is a rich source of new potential biomarkers, but these do not resolve easily with 2D gel electrophoresis (Issaq *et al*, 2002; Wang *et al*, 2003). Mass spectrometric technologies perform optimally in the low-molecular-weight range and early reports using surface-enhanced laser desorption and ionisation time-of-flight mass spectrometry (SELDI-TOF MS) have been promising (Bhattacharyya *et al*, 2004; Koopmann *et al*, 2004; Honda *et al*, 2005). Five groups have studied a serum protein panel using SELDI in combination with CA19-9 and have shown that such a combination of proteins is superior to CA19-9 alone (Koopmann *et al*, 2004; Honda *et al*, 2005; Ehmann *et al*, 2007; Guo *et al*, 2009; Navaglia *et al*, 2009).

Despite the similar findings of the above SELDI-based papers, there is no validated biomarker or biomarker panel for pancreatic adenocarcinoma (PC). Consequently, this paper describes an international study using SELDI-TOF MS to identify biomarkers that are followed by the use of ELISA to validate these on a further set of samples in which the disease controls (DCs) had severe pancreatic pathology. This work therefore aims to develop improved biomarkers that would be useful in the diagnosis of patients at an increased risk of pancreatic cancer.

## Materials and methods

### Clinicopathological details and sample collection

A total of 319 samples were obtained with patient consent. Training serum samples were obtained from 160 patients (the Training Set) managed at Centre-1 (Sydney, Australia) and their protein panels were validated against 80 plasma samples collected from subjects treated at Centre-2 (Verona, Italy; Validation-1 Set; Table 1). The samples were treated similarly with aliquots stored at −80 °C until analysis. Further confirmation of the findings was undertaken using ELISA methodology on a second set of 79 samples (Validation-2 Set), obtained from centre-1 from 33 PC patients, 28 DCs and 18 healthy volunteers (HVs). These controls were matched for age and sex. All DCs had pancreatico-biliary pathology requiring surgical or endoscopic intervention (Table 1).

Union Internationale Contre le Cancer (UICC) (Sobin, 2009) classification was used to stage the PC patients.

The study was approved by the ethics committees of the Northern Sydney Health Human Research, Sydney, Australia and the University of Verona, Verona, Italy.

### Preparation of serum or plasma for SELDI analysis

Serum or plasma was diluted 1 : 1 with denaturing buffer (8 M urea/1% CHAPS), and then centrifuged at 12 000 r.p.m. for 5 min). The supernatant was diluted 1 : 25 with trifluoroacetic acid (TFA), added to 50% acetonitrile/0.5% TFA, spotted on a hydrophobic (H50) protein chip array and processed as previously described (Scarlett *et al*, 2006). The protein chip arrays were analysed using the Bio-Rad Protein Biological System IIc ProteinChip Reader (Bio-Rad Hercules, CA, USA).

### SELDI-TOF MS analysis

Mass spectra were generated in the *m/z* range 2500–75 000 with a laser intensity setting of 220 (arbitrary units) and detector sensitivity set at 8. The laser was optimised for 4000–20 000 *m/z* peaks, whereas peaks <1000 *m/z* were deflected from the detector. Mean values from duplicate samples were used in subsequent analyses. The *m/z* value for each of the peaks was determined using external calibration with known standards (Sigma-Aldrich, St Louis, MO, USA): bovine insulin (5734.51 +1H), equine cytochrome *c* (12 361.96 +1H), equine apomyoglobin (16 952.27 +1H) and rabbit muscle aldolase (39 212.28 +1H). Spectra were analysed using the Ciphergen Protein Chip Software Version 3.1 (Bio-Rad).

### Protein purification and identification

The proteins of interest were size fractionated on a Superose 12 HR 10/300 GL column (Amersham Pharmacia Biotech, Uppsala, Sweden), equilibrated and eluted with 0.1 M acetic acid/0.1 M NaCl (pH 3.0) and fractions were monitored on SELDI using normal phase NP20 chips. Pooled fractions containing maximum activity were subjected to reverse-phase high-performance liquid chromatography (HPLC) on a 4.6 × 250 mm Jupiter 5 *μ*m, 300Å C18 column (Phenomenex, Torrance, CA, USA) after 30 min of gradient elution (15–60% acetonitrile in 0.1% TFA), and fractions were again monitored by SELDI-TOF MS on NP20 chips. The fraction containing the peaks of interest was lyophilised and then sent to the Bioanalytical Mass Spectrometry Facility (University of New South Wales, Sydney, Australia) for identification by tryptic peptide mass fingerprinting and MS sequencing. To confirm the protein identities, a SELDI immunoadsorption approach was performed. In this instance, a rabbit polyclonal Apolipoprotein C-I (ApoC-I) antibody (AbCam, Cambridge, UK) was bound to an RS100 protein chip array and analysed on the SELDI-TOF MS.

The three protein peaks at *m/z* 16 989, 17 132 and 17 247 were identified by a different strategy from above, following the report of Ehmann *et al* (2007), which described protein peaks at *m/z* 17 270 and 17 390 in human serum SELDI profiles as apolipoprotein A-II (ApoA-II) homodimers. The serum of patients suffering from PC and the purified human plasma ApoA-II (Sigma-Aldrich, Paris, France) were directly submitted to an RS100 preactivated chip coupled with ApoA-II antibody (AbCam, Paris, France).

### Western blotting validation

PC serum samples and purified human plasma ApoA-II were treated with 20 *μ*M dithiothreitol (DTT) from 0 to 4 h, respectively, and subjected to western blotting analysis with anti-ApoA-II antibody (AbCam) as described in a previous study (Xue *et al*, 2009).

### Statistical analysis

The raw peak intensity data were normalised using the total ion current between 2500 and 75 000 *m/z* and peak detection was performed using the Biomarker Wizard utility (Version 3.1, Bio-Rad). Sample group statistics were performed on peak intensity values for profiles of PCs *vs* DCs and HVs. Univariate analysis of individual peaks was performed using the non-parametric Mann–Whitney *U*-test with significance considered at *P*<0.05. The discriminatory power for each marker was characterised by receiver operating characteristic (ROC) area under the curve (AUC) analysis and the AUCs were compared using the Hanley and McNeil method (Hanley and McNeil, 1983; SPSS software Version 12.0, Chigaco, IL, USA). Values of ROC AUC are presented (Scarlett *et al*, 2006) with their 95% confidence intervals.

Co-correlation of protein peaks was examined by Spearman's non-parametric correlation coefficients because of the wide variance of the data for protein values, CA19-9 and bilirubin values.

### Development and validation of candidate biomarker models

#### Development

Biomarker panels were developed on the centre-1 serum cohort (*n*=160) using the multivariate binary logistic regression with ten-fold cross-validation technique previously described (Scarlett *et al*, 2006), which had been developed by Ambroise and McLachlan (Ambroise and McLachlan, 2002). Only serum peaks that significantly discriminated PCs from DCs or from HVs at *P*<0.01 were considered for multivariate analysis. This repeated random sampling procedure allowed for the correction of selection bias and enabled the calculation of unbiased estimates of sensitivity and specificity, overall accuracy and ROC AUC values with their 95% confidence intervals of the candidate biomarker panels.

#### Validation

The models developed in the training phase were then tested on the independent centre-2 plasma samples (*n*=80). Likelihood ratios (LRs) were calculated for each model to estimate the ratio of the likelihood of the test result in patients with disease to the likelihood of the same test result in patients without disease. Results with an LR of >10 or <0.1 effect a substantial change on disease likelihood over a broad range of pre-test estimates, whereas an LR of 1.0 leaves the likelihood of disease unchanged (Brown and Reeves, 2003).

### ELISA measurement of serum levels of CA19-9, ApoA-II and ApoC-I

Having developed the protein biomarker panel with SELDI, further confirmation was sought using ELISA as follows. Duplicate serum or plasma levels of CA19-9 were measured by ELISA kit (Alpha Diagnostic International, San Antonio, TX, USA) for which normal values were set at <37 U ml^–1^. Similarly, levels of ApoA-II and ApoC-I were measured by AssayMax ApoA-II ELISA kit and AssayMax ApoC-I ELISA kit (AssayPro, St Charles, MO, USA), respectively. Absorbance was measured at 450 nm on a microplate reader (Tecan, Salzburg, Austria) within 10 min.

## Results

### Patient characteristics

Three separate groups of subjects were studied. The initial training set from centre-1 consisted of 160 samples: 38 from PC patients (21 studied before surgery and 17 before palliative treatments), 54 DC samples from patients with other pancreatico-biliary disorders and 68 samples from HVs (Table 1). Although 18 of 39 PC and 10 of 54 DC patients had elevated serum bilirubin values, there was a significant difference between the PC and DC groups for mean bilirubin and other liver function test values (Table 2). The validation-1 samples from centre-2 were obtained from 40 PC subjects taken before resection, 21 DCs from patients requiring an acute admission and 21 HV subjects (Table 1). Of the PC patients from centre-2, 20 had elevated serum bilirubin values.

Validation-2 samples were from a further 79 patients from centre-1: 33 PC samples (21 from patients who underwent resection), 28 DC subjects (18 underwent pancreatic resection whereas 10 had therapeutic endoscopic retrograde cholangiopancreatography (ERCP)) and 18 HV subjects. The DCs and HVs had similar age and sex distribution (Table 1). In the validation-2 groups, bilirubin was elevated in 21 of 33 with PCs and 10 of 28 DCs (*χ*^2^=2.1, NS), but the median values were marginally different (*P*=0.047). The HVs had normal liver function values.

### Serum CA19-9 values of training and validation-1 samples

Blood CA19-9 levels were elevated in 63 of 78 PCs, 20 of 75 DCs and 15 of 85 HVs with greater mean (± s.e.m.) values in PC compared with DC and HV patients (CA19-9=1192.2 (±477.0), 34.3 (±5.0) and 19.7 U ml^–1^ (±2.1), respectively; *P*<0.001). Mean CA19-9 values were also higher in the stage IIb and IV patients (2212±1354 U ml^–1^) compared with the early stage I and IIa patients (163±19 U ml^–1^), but this was not statistically significant using the Mann–Whitney test.

### Development of diagnostic models from SELDI analysis

**Figure 1:**
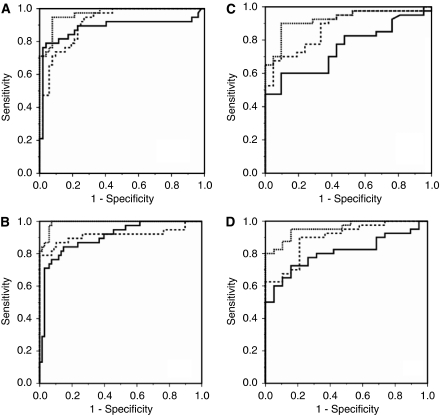
Receiver operator characteristic (ROC) curves for discrimination of pancreatic adenocarcinoma (PC) from disease controls (DCs) and healthy volunteers (HVs) in both the centre-1 and centre-2 cohorts. The protein panel derived from ten-fold cross-validation (solid line), CA19-9 (dashed line) and biomarker panel + CA19-9 (dotted line). (**A**) PC *vs* DC (centre-1): ROC AUC=0.90 for the biomarker panel, 0.87 for CA19-9 and 0.97 for the biomarker panel + CA19-9. (**B**) PC *vs* DC (centre-2): ROC AUC=0.88 for the biomarker panel, 0.75 for CA19-9 and 0.93 for the biomarker panel + CA19-9. (**C**) PC *vs* HV (centre-1): ROC AUC=0.90 for the biomarker panel, 0.91 for CA19-9 and 0.99 for the biomarker panel + CA19-9. (**D**) PC *vs* HV (centre-2): ROC AUC=0.90 for the biomarker panel, 0.81 for CA19-9 and 0.96 for the biomarker panel + CA19-9.

Differences in SELDI profiles were observed between PC, DC and HV subjects using the H50 protein chip array (Supplementary Figure S1). In all, 62 individual protein peaks observed in the *m/z* range 3000–20 000 were analysed. In the training set samples, 21 peaks were found to be differentially expressed between PCs and DCs. These individual putative tumour markers had ROC AUC values ranging from 0.62 to 0.81, with 14 upregulated in PC patients (Supplementary Table S1). Logistic regression with ten-fold cross-validation was applied to the subset of protein peaks that were differentially expressed between PCs and DCs to a significance of *P*<0.01 as determined by univariate analysis (Supplementary Table S1). This selected a panel of seven protein peaks (*m/z* 6420, 8451, 8614, 9137, 9626, 9694 and 12 862) that correctly classified 74% of PC and 87% of DC serum samples (ROC AUC: 0.90 (0.85–0.96), Supplementary Table S1 and Figure 1A). Discriminatory power was further improved by including the values of the tumour marker CA19-9 into the model. This model correctly classified 89% of PC and 96% of DC serum samples (ROC AUC: 0.97 (0.93–0.99), Supplementary Table S1 and Figure 1A).

**Figure 2:**
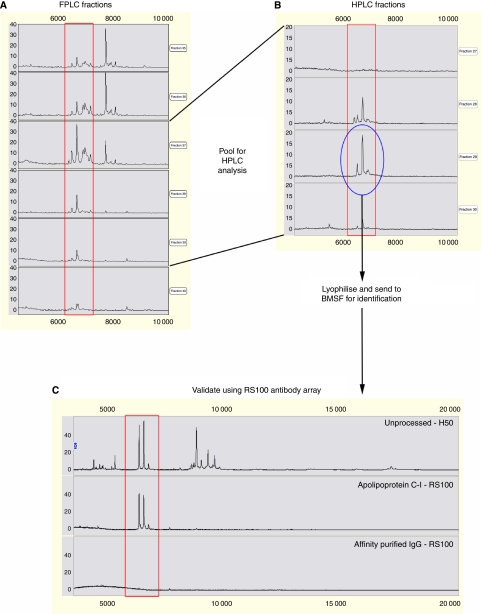
Purification, identification and validation of the apparent 6.6 kDa proteins from the serum of a patient with pancreatic adenocarcinoma. (**A**) Proteins from serum were run through a Superose 12 HR 10/300 GL column, eluted with 0.1 M acetic acid/0.1 M NaCl (pH 3.0) and fractions were monitored by SELDI-TOF MS using NP20 chips. (**B**) Pooled fractions were further purified by reverse-phase HPLC with a 40 min 15–60% acetonitrile gradient in 0.1% trifluoroacetic acid and fractions were monitored by SELDI-TOF MS using NP20 chips. The fraction containing the 6.6 kDa proteins (circled) was then lyophilised and sent to BMSF for identification. (**C**) Identification of the 6.6 kDa proteins was validated using a SELDI immunoassay approach (RS100 antibody array).

When the protein peak model was applied to the validation-1 samples, 90% of PC and 67% of DC samples were correctly classified (ROC AUC: 0.88 (0.79–0.96), Supplementary Table S1 and Figure 1B). When CA19-9 was included in the model, 93% of PC and 71% of DC samples were correctly classified (ROC AUC: 0.93 (0.86–0.99)). For both the training and validation-1 cohorts, the ROC AUC values were significantly increased by the addition of CA19-9 (*P*<0.05) and were significantly greater than that for CA19-9 alone (Figures 2C and D).

### PC *vs* HV serum

In all, 18 peaks were found to be differentially expressed between PCs and HVs, 13 with a *P-*value ⩽0.01, using logistic regression analysis (Supplementary Table S1). The ten-fold cross-validation approach selected a final panel of four peaks (*m/z* 6618, 16 989, 17 132 and 17 247) that correctly classified 71% of PC and 96% of HV serum samples (ROC AUC: 0.90 (0.84–0.96), Supplementary Table S1 and Figure 1C), which was similar to the AUC of CA19-9 (ROC AUC: 0.914 (0.839–0.989)). When CA19-9 was added to the protein panel, the discriminatory power was better (*P*<0.05) than for the protein panel or CA-19-9 alone – 92% of PC and 97% of HV samples were correctly classified (ROC AUC: 0.99 (0.98–1.00)).

When the logistic regression equation from the training set was applied to the validation-1 sample set, 90% of PC and 74% of HV serum samples were correctly classified (ROC AUC: 0.90 (0.81–0.98), Supplementary Table S1 and Figure 1D). Again, the addition of CA19-9 significantly improved (*P*<0.05) the classification, identifying 93% of PC and 84% of HV samples (ROC AUC: 0.96 (0.91–1.00)), which was greater than that for CA19-9 alone (ROC AUC: 0.81 (0.61–0.92), but this did not reach significance.

### Testing for co-correlation of the protein values

Spearman's correlation coefficients for the variables selected in the above protein panels (Supplementary Table S2) indicate that these proteins were associated with one of two groups by close correlation. One group was closely correlated with the *m/z* 6420 peak and the others were closely correlated with the *m/z* 17 247 peak. Subsequent analysis of the identities of these peaks clarified this correlation.

### Protein purification and identification

Serum samples that showed high *m/z* 6420 and 6618 peak intensities were purified for identification of the apparent 6.6 kDa proteins. The protein fractions were monitored on SELDI using NP20 chips (Figure 2A). Pooled fractions containing maximum activity were further purified using HPLC and again monitored on NP20 chips (Figure 2B). The fractions containing the peaks of interest were lyophilised for identification by tryptic peptide mass fingerprinting and MS sequencing. The *m/*z 6618 peak was identified as ApoC-I from three peptides that covered 38.5% of the amino acid sequence. To confirm the protein identities obtained from sequencing, a SELDI immunoadsorption approach was performed using an ApoC-I antibody bound to an RS100 protein chip array. The *m/z* 6420 and 6618 peaks were captured with high intensity with minimal nonspecific binding observed for the affinity-purified IgG control (Figure 2C).

**Figure 3:**
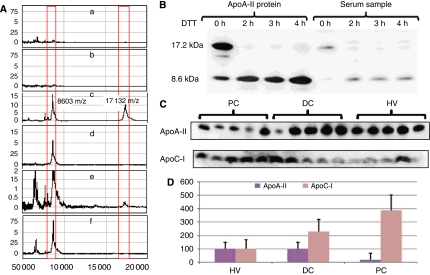
(**A**) Plots of peak height against *m/z* for the identification of ApoA-II isoforms using SELDI RS100 profiles. (a) ApoA-II purified human plasma ApoA-II (Sigma-Aldrich) IgG, (b) serum without IgG, (c) purified human ApoA-II on ApoA-II antibody-enriched chip indicating a number if peaks ∼8603 indicating monomer and 17 132 indicating dimer, (d) purified ApoA-II treated with 20 *μ*M DTT for 3 h on antibody-enriched chip, (e) serum on ApoA-II antibody-enriched chip and (f) purified ApoA-II treated with 20 *μ*M DTT for 3 h on antibody-enriched chip. (**B**) Western blotting analysis of the effect of 0 to 4 h of 20 *μ*M DTT reduction on a serum sample and on purified human plasma ApoA-II. (**C**) Western blot of the expression of ApoC-I and ∼17 kDa ApoA-II in five pancreatic cancer samples (PC), five disease control samples (DC) and five human volunteer samples (HV). (**D**) Plot of mean and s.d. band intensity (% of HV) as semiquantified by Pro-Gel scanning (Gel-Pro Analyzer Properties) for PC samples (*n*=15), DC samples (*n*=15) and HV samples (*n*=15).

The *m/z* 8614, 16 989, 17 185 and 17 247 proteins were confirmed as ApoA-II isoforms by examining samples of serum and purified human ApoA-II. Human ApoA-II not only showed the appearance of three major isoforms, *m/z* 16 989, 17 185 and 17 247, but also of a peak at 8614. A similar pattern was observed in the plasma specimens (Figure 3 Ae) in SELDI MS profile. Using SDS–PAGE according to Gillard *et al* (2005), specimens of purified ApoA-II and serum samples from PC patients were reduced for 3 h using 20 *μ*M DTT. This resulted in a loss of the 17-kDa band and an increase in the 8.6-kDa band, which is consistent with reduction of disulfide-linked homodimers.

The PC serum samples and purified human plasma ApoA-II were treated with 20 *μ*M DTT from 0 to 4 h. The reduced samples were then submitted to western blotting analysis with anti-ApoA-II antibody. Figure 3B shows that both crude serum and purified ApoA-II contained the ApoA-II dimer (∼17.2 kDa) and the bands disappeared after reduction by DTT. Furthermore, the band at 8.6 kDa was concomitantly increased in intensity upon DTT treatment. Figures 3C and D show the differences in concentration of ApoC-I and ApoA-II in non-reducing conditions according to Gillard *et al* (2005) on western blot and the semiquantitative expression undertaken in 15 serum samples from each of the PC, DC and HV subjects.

### Simplified diagnostic panel

**Figure 4:**
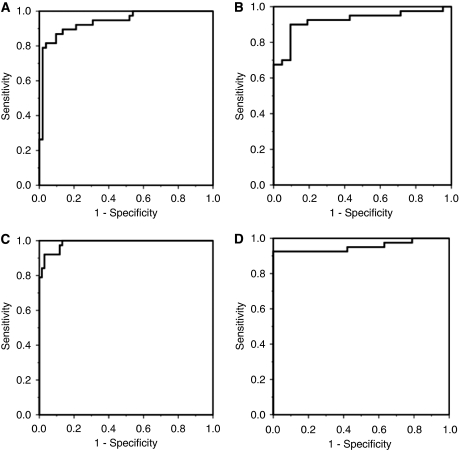
ROC curves for the performance of the simplified biomarker panel (CA19-9 and the protein peak heights for *m/z* 6420 (ApoC-I) and 17 247 proteins (ApoA-II)). (**A–D**) PC *vs* DC for centre-1 samples, PC *vs* DC for centre-2 samples, PC *vs* HV for centre-1 samples and PC *vs* HV for centre-2 samples are shown (see text for AUC values).

Because of the high correlation coefficients between all of the proteins in the diagnostic panels to either ApoC-I or ApoA-II, a simplified diagnostic panel was proposed combining the ApoC-I *m/z* 6420 peak and the ApoA-II *m/z* 8614 with CA19-9. This combination was used to examine the diagnostic effectiveness in the four different patient groups and showed strong ROC AUC values of 0.99 for PC *vs* HV in the training set, 0.95 for PC *vs* HV in the validation-1 set, 0.94 for PC *vs* DC in the training set and 0.92 for PC *vs* DC subjects in the validation-1 set (Figures 4A–D). The positive and negative LRs for the above comparisons were 16.5, 5.4, 6.7 and 4.7 and 0.06, 0.11, 0.07 and 0.19, respectively, indicating that this test would make a useful contribution to clinical decision making but would not be useful for screening purposes.

### ELISA validation

**Figure 5:**
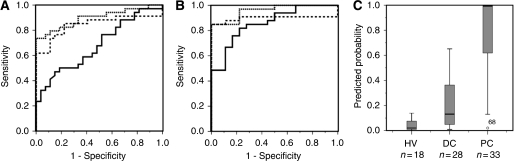
ROC curves for the ELISA measures on the validation-2 set. (**A**) PC *vs* HV and (**B**) PC *vs* DC. Dotted lines represent the curves for combined ApoA-II, ApoC-I and CA19-9, dashed lines indicate CA19-9 whereas solid lines indicate the combined protein panel of ApoA-II and ApoC-I (see text for AUC values). (**C**) The probability of diagnosing PC as separate from benign cases (combined HV and DC) using binary logistic regression on the ELISA values of ApoA-II, ApoC-I and CA19-9.

ELISA mean (s.e.m.) measurements on the HV, DC and PC validation-2 set for ApoA-II were 55 (2), 47 (1) and 44 g l^–1^ (1) and for ApoC-I were 107 (4), 101 (6) and 124 g l^–1^ (12). For ApoC-1 the difference between DCs and PCs indicated a trend to significance at *P*=0.07 and the mean ApoA-II values were different between HVs and PCs (*P*=0.001), whereas the mean differences between DCs and PCs were not significant. Although there were significant differences between the PC and DC patients in the values of bilirubin and other liver function tests, these were less significant than those from the training sets (Table 2). ROC analysis confirmed a significant diagnostic influence of the combination of two proteins, ApoA-II and ApoC-I, with AUC of 0.86 (0.76–0.96) for PC *vs* HV and 0.68 (0.55–0.81) for PC *vs* DC patients. The addition of these proteins to CA19-9 improved the ROC AUC compared with CA19-9 alone to 0.96 (0.90–1.0) *vs* 0.90 (0.80–0.99) for HV samples and 0.90 (0.82–0.98) *vs* 0.84 (0.74–0.95) for DC samples (Figure 5). Multivariate stepwise backward analysis showed significance for the combined panel but no influence from the addition of bilirubin, albumin or serum alkaline phosphatase was observed. Figure 5C shows the probability of diagnosing pancreatic cancer compared with benign disease (HV and DC cases combined), derived from binary logistic regression using the ELISA values for ApoA-II, ApoC-I and CA19-9.

## Discussion

This international collaborative study shows the utility of SELDI-TOF MS for identification of potential diagnostic biomarker panels that have been confirmed on two independent sample sets. The significant proteins in the diagnostic panel were identified as ApoA-II and ApoC-I. This allowed for a simplified panel, the efficacy of which was confirmed on validation-2 samples by ELISA. Although this study has similar motives to previous studies using SELDI-TOF MS (Koopmann *et al*, 2004; Honda *et al*, 2005; Ehmann *et al*, 2007; Guo *et al*, 2009; Navaglia *et al*, 2009), important new findings are described. Both ApoC-I (Takano *et al*, 2008) and ApoA-II (Ehmann *et al*, 2007) have been identified in previous studies, but they have not been used in combination, in which they seem to provide independent contributions to the diagnostic panel. The sensitivities and specificities of new individual peaks were similar to that of CA19-9 in discriminating PCs from both DCs and HVs, but importantly discriminatory power was significantly improved by multi-protein marker models that included CA-19-9. The robustness of the model was shown by the fact that although developed on a training set of serum samples, it was able to be validated on plasma samples (Banks *et al*, 2005). Further support for the diagnostic capacity of this protein panel was confirmed by ELISA on a separate sample set.

Both the PC *vs* DC and PC *vs* HV classification models improved the discriminatory power when compared with CA19-9 alone. The importance of CA19-9 as a diagnostic marker of pancreatic cancer (Goonetilleke and Siriwardena, 2007) is confirmed by this study, but its discriminating ability is significantly improved by the SELDI-derived diagnostic panels. Furthermore, the overall accuracy of the combined results indicates that these diagnostic models may become clinically useful in high-risk patient groups. However, because of the low prevalence of pancreatic cancer, a much greater accuracy would be required before these diagnostic panels could be clinically useful in an asymptomatic population (Bhattacharyya *et al*, 2004).

ApoA-II was observed in both the monomeric form of *m/z* 8614 and in the dimers of *m/z* 16 989, 17 185 and 17 247 in the SELDI MS profiles. In human plasma or serum, ApoA-II mainly exists as a disulfide-linked homodimer at approximately 17.3 kDa (Gillard *et al*, 2005). There seems to be a number of isoforms that may account for the smaller adjacent peaks of *m/z* 16 989 and 17 132. The apolipoproteins lie on the surface of lipid particles and have an important role in directing the fate of these particles to different organs for metabolism. The precise function of ApoA-II is not entirely clear but it is suggested that ApoA-II is rich in HDL particles that promote the formation of atheroma (Stein *et al*, 1995). The exact metabolism of ApoA-II is not completely known. It has a diagnostic role in prostate carcinoma that has been shown to release ApoA-II (Malik *et al*, 2005). In comparison with pancreatic cancer, in which low levels are diagnostic, in prostate cancer high levels are diagnostic.

Five research groups using SELDI-TOF for the identification of serum markers of pancreatic cancer have also developed multiple marker panels that include CA19-9 (Koopmann *et al*, 2004; Honda *et al*, 2005; Ehmann *et al*, 2007; Guo *et al*, 2009; Navaglia *et al*, 2009). Koopmann *et al* (2004) used IMAC-Cu^2+^ and weak cationic exchange protein chips and found a two-peak panel that differentiated PC samples from HVs, whereas a three-peak panel distinguished PCs from DCs. These panels performed better than CA19-9 alone, but when used in combination, the diagnostic accuracy was further improved. Guo *et al*, (2009) used strong anion-exchange protein chips to show that the three protein biomarkers, *m/z* 4155, 4791 and 28 068, are more accurate than CA19-9 in differentially diagnosing pancreatic cancer. The larger peak was identified as the protein C14orf16 that is highly expressed in cancer tissue, although the function of this protein is not fully elucidated. Yu *et al* (2005) used the Biomarker Patterns software (Bio-Rad) to develop a classification tree that includes six peaks that differentiate cancer from non-cancer with high sensitivity and specificity without CA19-9 in 100 subjects, using IMAC-Cu^2+^ protein chips, and recently this group has shown high sensitivity and specificity of the protein panel. Honda *et al* (2005) used three protein chip types (IMAC-Cu^2+^, H50 and CM10) on 24 subjects, and developed training models using a support vector machine algorithm that selected four peaks, three from the CM10 protein chip and one from the H50, which when combined could distinguish pancreatic cancer from healthy controls with high sensitivity and specificity. The SELDI-derived panel was superior to that of CA19-9 alone, but was further improved in combination with CA19-9. A fifth recent study compared serum from cancer patients and healthy volunteers and showed that a panel of three proteins had a sensitivity of 83% and a specificity of 96% when tested in an independent validation set of samples. Interestingly, ApoA-II was identified, along with ApoA-I and transthyretin (Ehmann *et al*, 2007). These proteins are produced by the liver and have important roles in lipid transport, and although their full function is not understood, they are components of HDL. Although these studies report differing numbers and peaks that constitute the diagnostic panels that show promise, each lacks the identification of many of the proteins of interest.

Our findings that lipoproteins are perturbed in pancreatic caner suggest that lipid metabolism is important for such tumours. This is supported by cell culture studies that show greater cell growth in the presence of lipid (Wang *et al*, 2009). Therefore, it is interesting that the apolipoproteins seen to be markers of pancreatic cancer are both involved with lipid transport. ApoA-II is a component of HDL subclass 3 in which it combines with ApoA-I and results in a smaller particle size by increasing its hydrophobic properties (Gao *et al*, 2009). This acts as a counter to the cardio-protective effects of ApoA-I. However, the exact function of ApoA-II is unclear (Blanco-Vaca *et al*, 2001; Gao *et al*, 2009). It is possible that ApoA-II helps direct lipid to the cancer. However, there are a number of alternative reasons that the concentration of apolipoproteins may be depressed in patients with pancreatic cancer: weight loss (Ng *et al*, 2010), liver dysfunction (Tacikowski *et al*, 2000) and diabetes (Onat *et al*, 2009). It will be important to study this further to determine whether this effect is directly related to PC demand for nutrition or whether they are an epiphenomenon of the weight loss associated with the cancer.

This study found that the *m/z* 6618 peak, identified as ApoC-I, was most frequently selected for the PC *vs* HV model by ten-fold cross-validation. Investigation of the serum/plasma MS literature revealed that ApoC-I exists in human serum as ApoC-I (∼6630 Da) and a truncated isoform that lacks N-terminal Thr-Pro- (6432 Da) (Jin and Manabe, 2005). ApoC-I has an important role in controlling plasma lipid metabolism, but little is known about its role in the cancer process (Bondarenko *et al*, 1999). Genetic upregulation of ApoC-I has been shown in gastric cancer (Oue *et al*, 2004), whereas in a recent breast cancer study ApoC-I formed part of a multi-protein index (developed from SELDI analysis) that could predict metastatic relapse in high-risk primary breast cancer patients receiving adjuvant chemotherapy (Goncalves *et al*, 2006). ApoC-I has also been shown in pancreatic cancer and is suggested to be associated with infiltrating macrophages within the juxtatumoural stroma (Ricci *et al*, 2005). Ricci *et al* (2005) suggested that this may be an indication of direct communication between stroma and cancer cells and provides evidence of a response to infiltrative growth that may predominate in tumour–stromal interactions independent of cancer type.

The inflammatory process associated with cancer may have important prognostic implications (Pine *et al*, 2009) and the specificity of this may provide opportunities for biomarker discovery. Using electrospray ionisation (ESI) ion-trap tandem MS (ESI-MS) to explore the differences between human pancreatic cancer sera and normal sera, Hanas *et al* (2008) revealed greater heterogeneity in cancer sera, especially in the low-mass region. Using a statistical bootstrap approach, they showed that three large-mass proteins involved in inflammatory responses were elevated in pancreatic cancer sera: *α*-2 macroglobulin, ceruloplasmin and complement 3C. Firpo *et al* (2009) also improved the discriminatory power of CA19-9 when used with two acute-phase proteins: haptoglobin and serum amyloid A. In our validation-2 data, ApoC-I levels correlated with white cell count; although ApoC-I is an inflammatory marker, the results confirm those of Takano *et al* (2008) that ApoC-I is also a potentially useful marker for pancreatic cancer.

This international collaborative study confirms the usefulness of SELDI-TOF MS for exploration of low-mass proteome (Guo *et al*, 2009) in which there are important signatures of the cancer process (Navaglia *et al*, 2009) when PC is resectable. Because of the complex cancer process, a panel of biomarkers is likely to be useful in high-risk groups of patients. Further exploration of the exact function of these proteins may provide insights into PC. It will be important to discover how early in the tumourigenic process these proteins become altered, and whether these panels of proteins are capable of diagnosing curable PC.

## Figures and Tables

**Table 1:** Detail of patient groups

	**Training SELDI**	**Validation-1 SELDI**	**Validation-2 ELISA**
	***n***	***n***	***n***
*Pancreatic ductal adenocarcinoma*	38	40	33
Male	20	23	15
Female	18	17	18
Stage I	7	—	1
Stage IIa	5	4	7
Stage IIb	8	36	14
Stage IV	18	—	11
			
*Disease control*	54	21	28
Male	28	18	16
Female	26	3	12
Cholelithiasis	17	2	
Choledocholithiasis			6
Intraductal papillary mucinous neoplasm	6	—	1
Carotid artery stenosis	—	6	
Mucinous cystadenoma	5	—	2
Neuroendocrine (islet cell) tumour	5	—	7 (4)
Gallstone pancreatitis	4	—	2
Chronic pancreatitis	4	1	2
Benign stricture			2
Hernia	3	2	
Acute cholecystitis	—	3	
Other^a^	10	7	3
			
*Healthy volunteer*	68	19	18
Male	28	12	9
Female	40	7	9

Abbreviation: SELDI=surface-enhanced laser desorption and ionisation.

The number in brackets indicates malignant islet cell tumours.

a Other includes: Training Set – villous adenoma (*n*=2), serous cystadenoma (*n*=2), pancreatic pseudocyst (*n*=2), solid pseudopapillary tumour (*n*=1), Caroli's disease (*n*=1), gastro-oesophageal reflux (*n*=1) and ruptured appendix (*n*=1); Validation-1 Set – leg ischaemia (*n*=2), rectal bleeding (*n*=2), diaphragmatic hernia (*n*=1), phlebitis (*n*=1) and haematological disorder (*n*=1); and Validation-2 Set – solitary fibrous tumour, granulomata, intrapancreatic pseudocyst.

**Table 2:** Mean and s.e.m. of age and biochemical indices for patient groups

		**Pancreatic cancer**		**Disease controls**		***P*-value**
**Values**	**Set**	**Mean**	**s.e.m. or range**	**Mean**	**s.e.m. or range**	**PC *vs* DC**
Age (years)	Training	70.9	(28–80)	62.7	(32–81)	NS
	Validation-2	69.7	(32–81)	57.1	(18–89)	NS
Urea (mmol l^–1^)	Training	5.9	0.5	5.1	0.4	NS
	Validation-2	5.8	0.4	5.6	0.6	NS
Bilirubin (*μ*mol l^–1^)	Training	144	33	18	3	0.001
	Validation-2	88	20	49	21	0.01
Albumin (g l^–1^)	Training	35.1	1.2	41.1	0.8	0.001
	Validation-2	35.5	1.1	39.4	1.1	0.017
AST (IU l^–1^)^a^	Training	111	19	50	10	0.001
	Validation-2	98	19	53	9	0.049
ALT (IU l^–1^)^a^	Training	154	28	76	21	0.005
	Validation-2	143	32	94	26	NS
ALP (IU l^–1^)^a^	Training	362	60	101	14	0.001
	Validation-2	316	45	172	23	0.007
GGT (IU l^–1^)^a^	Training	489	102	130	43	0.001
	Validation-2	561	111	208	49	0.006
CA19-9 (U l^–1^)^a^	Training	1594	960	32	7	0.001
	Validation-2	1466	922	31	9	0.001

Abbreviations: ALP= alkaline phosphatase; AST= glutamic-oxaloacetic transaminase; ALT= glutamic-pyruvic transaminase; CA19-9= carbohydrate antibody 19-9; DC= disease control; GGT= *γ*-glutamyltransferase; NS= not significant; PC= pancreatic cancer.

a Results are for serum values.

*P-*value indicates Mann–Whitney *U*-test result.
