# Supplementary material for: Discovery of serum biomarkers for pancreatic adenocarcinoma using proteomic analysis
Source: Br J Cancer. 2010 Jun 29;103(3):391–400. doi: 10.1038/sj.bjc.6605764 (PMC2920018; doi:10.1038/sj.bjc.6605764)
Supplement: Supplementary Figure S1 [file 6605764x1.doc]

**
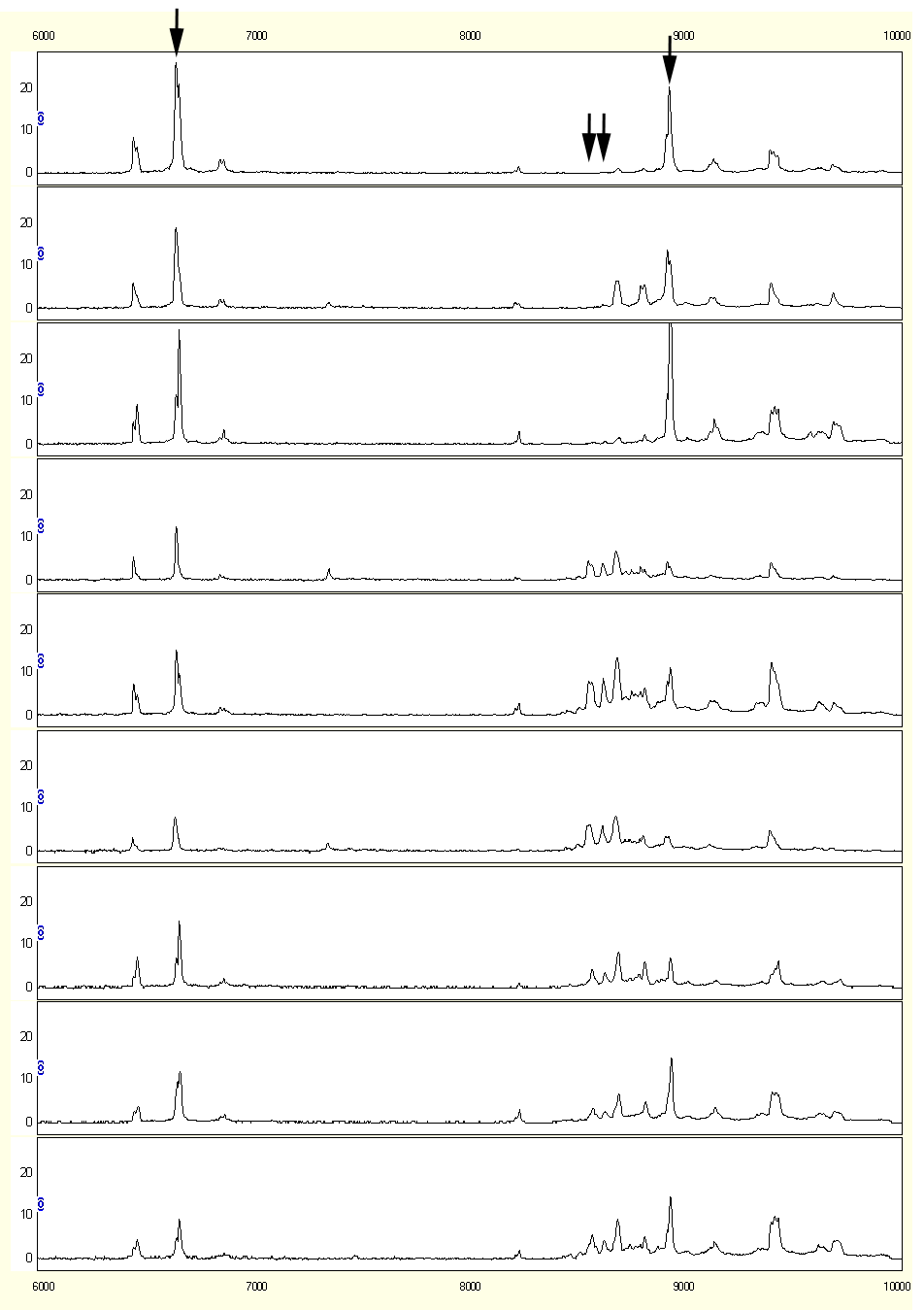
**

**PC**

**HV**

**DC**

**Figure S1:** A segment of the protein mass profile between *m/z* 6000 and 10000 demonstrating differentially expressed peaks between serum from pancreatic adenocarcinoma (PC), healthy volunteers (HV) and disease control (DC) patients. Arrows indicate differentially expressed peaks (Mann Whitney U Test).
