# Supplementary material for: Discovery of serum biomarkers for pancreatic adenocarcinoma using proteomic analysis
Source: Br J Cancer. 2010 Jun 29;103(3):391–400. doi: 10.1038/sj.bjc.6605764 (PMC2920018; doi:10.1038/sj.bjc.6605764)
Supplement: Supplementary Table S1 [file 6605764x2.doc]

***Table S1:*** *Protein peaks differentially expressed between pancreatic adenocarcinoma (PC) v. disease control (DC) and healthy volunteer (HV) serum in “Training cohort”.*

***Results for PC v. DCResults for PC v. HVm/zROC AUCP valuem/zROC AUCP value***38790.640.023^a^38790.620.046^a^41020.620.049^a^45570.640.014^a^45570.650.013^a^64200.650.013^a^46990.640.026^a^66180.680.002^a,b^64200.660.008^a,b^84510.75<0.00166180.710.001^a^85580.86<0.00179230.640.028^a^86140.82<0.00184510.77<0.001^b^87490.660.00585580.81<0.00189160.660.008^a^86140.70.001^b^91370.650.010^a^89160.72<0.001^a^96940.660.007^a^91370.73<0.001^a,b^99160.650.010^a^94220.650.013^a^125610.620.0496260.660.010^a,b^128620.630.029^a^96940.74<0.001^a,b^169890.8<0.001^b^99160.75<0.001^a^170620.8<0.001128620.690.002^a,b^171320.87<0.001^b^169890.81<0.001172470.84<0.001^b^170620.79<0.001   171320.81<0.001   172470.690.002   *m/z* is mass to charge of individual proteins.

^a^ Increased expression in pancreatic adenocarcinoma samples following univariate analysis.

^b^ Peaks included in the candidate biomarker training models following logistic regression and 10-fold cross validation.

.

^b^Peaks included in the candidate biomarker training models following logistic regression and 10-fold cross validation. The numbers in superscript indicate the number of times this protein was identified by the 10 fold technique
