# Supplementary material for: Discovery of serum biomarkers for pancreatic adenocarcinoma using proteomic analysis
Source: Br J Cancer. 2010 Jun 29;103(3):391–400. doi: 10.1038/sj.bjc.6605764 (PMC2920018; doi:10.1038/sj.bjc.6605764)
Supplement: Supplementary Table S2 [file 6605764x3.doc]

**Table S2. Spearman’s correlation coefficients for non parametric correlation between proteins of interest, serum bilirubin and albumin:**

| **Correlation coefficients for Spearman's Correlation** | | | | | | | | | | | |
| --- | --- | --- | --- | --- | --- | --- | --- | --- | --- | --- | --- |
|  | P6420 | P6618 | P17132 | P17247 | P16989 | P8614 | P8451 | P9137 | P9626 | P9694 | P12862 |
| Bilirubin | 0.39 | 0.45 | -0.36 | -0.44 | -0.43 | -0.44 | -0.22 | 0.37 | 0.21 | 0.14 | 0.02 |
| Albumin | -0.03 | -0.21 | 0.23 | 0.27 | 0.21 | 0.27 | 0.18 | -0.32 | -0.29 | -0.3 | -0.35 |
| CA 19-9 | 0.14 | 0.28 | 0.16 | 0.01 | 0.16 | -0.01 | 0.471^*^ | 0.23 | 0.24 | 0.22 | 0.17 |
| *m/z*6420 |  | 0.88** | 0.42 | 0.24 | 0.27 | 0.25 | 0.1 | 0.66** | 0.55** | 0.66** | 0.52* |
| *m/z*6618 |  |  | 0.25 | 0.1 | 0.15 | 0.11 | -0.01 | 0.69** | 0.54* | 0.62** | 0.43* |
| *m/z*17132 |  |  |  | 0.92** | 0.88** | 0.94** | 0.56** | 0.33 | 0.46* | 0.41 | 0.59** |
| *m/z*17247 |  |  |  |  | 0.85** | 0.98** | 0.49* | 0.24 | 0.37 | 0.28 | 0.42* |
| *m/z*16989 |  |  |  |  |  | 0.82** | 0.63** | 0.16 | 0.29 | 0.26 | 0.36 |
| *m/z*8614 |  |  |  |  |  |  | 0.48* | 0.26 | 0.37 | 0.28 | 0.46* |
| *m/z*8451 |  |  |  |  |  |  |  | 0.1 | 0.15 | 0.15 | 0.19 |
| *m/z*9137 |  |  |  |  |  |  |  |  | 0.83** | 0.80** | 0.55** |
| *m/z*9626 |  |  |  |  |  |  |  |  |  | 0.85** | 0.57** |
| *m/z*9694 |  |  |  |  |  |  |  |  |  |  | 0.57** |
| * Correlation is significant at the .01 level (2-tailed).  ** Correlation is significant at the .000 level (2-tailed). | | | | | |  |  |  |  |  |  |
|  |  |  |  |  |  |
